# Chemopreventive Effect of the Germinated Oat and Its Phenolic-AVA Extract in Azoxymethane/Dextran Sulfate Sodium (AOM/DSS) Model of Colon Carcinogenesis in Mice

**DOI:** 10.3390/foods9020169

**Published:** 2020-02-10

**Authors:** Margarita Damazo-Lima, Guadalupe Rosas-Pérez, Rosalía Reynoso-Camacho, Iza F. Pérez-Ramírez, Nuria Elizabeth Rocha-Guzmán, Ericka A. de los Ríos, Minerva Ramos-Gomez

**Affiliations:** 1 Research and Graduate Studies in Food Science, School of Chemistry, Autonomous University of Queretaro, Cerro de las Campanas S/N, Querétaro 76010, Mexico; dalima06@gmail.com (M.D.-L.); rrcamachomx@yahoo.com.mx (R.R.-C.); iza.perez@uaq.mx (I.F.P.-R.); 2 School of Natural Sciences, Autonomous University of Queretaro, Av. de las Ciencias S/N, Juriquilla, Querétaro 76230, Mexico; gperosas8@gmail.com; 3 TECNM/Instituto Tecnológico de Durango, Felipe Pescador 1830 Ote., Durango 34080, Mexico; 4 Institute of Neurobiology, Universidad Nacional Autónoma de México (UNAM), Campus UNAM-Juriquilla, Querétaro 76230, Mexico; eridie9dic@hotmail.com

**Keywords:** germinated oat, avenanthramides, colorectal cancer, chemoprevention

## Abstract

The consumption of fruits, vegetables, nuts, legumes, and whole grains has been associated with a lower risk of colorectal cancer (CRC) due to the content of natural compounds with antioxidant and anticancer activities. The oat (*Avena sativa* L.) is a unique source of avenanthramides (AVAs), among other compounds, with chemopreventive effects. In addition, oat germination has shown enhanced nutraceutical and phytochemical properties. Therefore, our objective was to evaluate the chemopreventive effect of the sprouted oat (SO) and its phenolic-AVA extract (AVA) in azoxymethane (AOM)/dextran sulfate sodium (DSS)-induced CRC mouse model. Turquesa oat seeds were germinated (five days at 25 °C and 60% relative humidity) and, after 16 weeks of administration, animals in the SO- and AVA-treated groups had a significantly lower inflammation grade and tumor (38–50%) and adenocarcinoma (38–63%) incidence compared to those of the AOM+DSS group (80%). Although both treatments normalized colonic GST and NQO1 activities as well as erythrocyte GSH levels, and significantly reduced cecal and colonic β-GA, thus indicating an improvement in the intestinal parameters, the inflammatory states, and the redox states of the animals, SO exerted a superior chemopreventive effect, probably due to the synergistic effects of multiple compounds. Our results indicate that oats retain their biological properties even after the germination process.

## 1. Introduction

Colorectal cancer (CRC) is the third most diagnosed malignancy and the fourth leading cause of cancer death around the world, and its burden is expected to increase by 60% to more than 2.2 million new cases and 1.1 million cancer deaths between now and 2030 [1]. CRC presents as a series of genetic and morphological changes in the colonic epithelium, which begins with the formation of aberrant crypt foci (ACF), followed by polyps and adenomas, until the development of adenocarcinomas occurs [2]. The etiology of this disease is diverse; however, there are two main risk factors associated: 80–90% of CRC cases are due to environmental factors such as diet and lifestyle, while only 10–20% are due to hereditary factors or genetic alterations. In this regard, diets high in saturated fat and the consumption of processed red meats along with diets low in vegetable and cereal intake increase the risk of CRC [3,4,5].

Cereals constitute the main source of food worldwide; they are also considered to have high nutritional value, as they contain several beneficial elements, such as starch, proteins, fiber, lipids, and phytochemicals [6]. Oats are an important source of livestock feed worldwide, both as forage and as a nutritious grain. Like all other cereal grains, oats belong to the Pomaceae family (also known as the Gramineae). *Avena sativa* L. (common oat) is considered the most important species among cultivated oats [7]. In this sense, oats of the Turquesa variety, derived from a cross with the Karma variety, have the characteristics of high adaptation, yield stability, and disease-resistance [8].

In recent years, oats have attracted growing attention as a health food, involved in a lower risk of cardiovascular diseases (CVD), type 2 diabetes mellitus (T2DM), gastrointestinal disorders, and cancer [9]. Those properties have been attributed to their content of various bioactive compounds, such as β-glucan, the main component of the soluble fiber in oats. There is evidence revealing that soluble and insoluble β-glucan exerted favorable effects in preventing colon cancer in a dose-dependent manner, although the specific mechanism might be different [10]. Furthermore, oat β-glucan exhibited an anti-inflammatory effect against colitis through inhibition of expression of pro-inflammatory factors in a DSS-induced ulcerative colitis model [11]. In addition, oats have been associated with the presence of several antioxidant compounds, such as tocopherols and tocotrienols, which have the ability to act as strong free-radical scavengers, also showing the capacity to inhibit the proliferation of malignant colon cancer cells [12]. The inhibitory effects of avenacosides (oat-unique steroidal saponins) against the growth of human colon cells has also been evaluated through diverse mechanisms, such as the inhibition of tumor cell growth by cell cycle arrest and the stimulation of apoptosis, among others [9]. Moreover, phenolic compounds, such as ferulic acid and avenanthramides (AVA), are the most abundant components in oats and have documented antioxidant, anti-inflammatory, and antiproliferative activities [13,14]. In vitro studies indicate that AVAs prevent cancer mainly by blocking reactive species, and exhibit potential therapeutic activity through the modulation of different pathways, including the activation of apoptosis and senescence and the blocking of cell proliferation. In this context, AVA-A (2p) notably attenuated tumor formation in an azoxymethane (AOM)/dextran sulfate sodium (DSS) model, most likely through the induction of cellular senescence [15].

On the other hand, cereal sprouts have received considerable attention as functional foods in many countries, especially in Europe, the United States of America, and Japan, due to their superior nutritious and health benefits compared to seeds [16]. The germination of cereals has been used for centuries to soften kernel structure, decrease the content of antinutritive compounds, and to increase nutrient content and availability [17]. In this regard, an increase in the nutraceutical and phytochemical profile of oats could improve their biological activities, such as in the case of AVA-C (2c) from germinated oats, which exhibited antiallergic and anti-inflammatory activities [18]. Additionally, numerous trials and animal studies have demonstrated the bioavailability and bioaccessibility of polyphenolic compounds of oats, especially AVAs, and their association with an improved antioxidant status [19,20,21,22]. Similarly, mounting evidence highlights that the absorption, bioavailability, and metabolism of several phytochemicals is a crucial factor in determining their biological activity against colon cancer [23]. Therefore, the metabolism of dietary flavonoids in the digestive tract by the gut microbiota is important for increasing their bioavailability and determining their impact on our health.

To the best of our knowledge, only a few studies have focused on the relationship between an enhanced phytochemical profile and the biological values of cereal sprouts, and there is currently not enough available information regarding the chemopreventive activity of germinated oats. Therefore, the purpose of this investigation was to evaluate whether the sprouted oat (SO) of the Turquesa variety still possessed effective physiologically bioactive compounds, i.e., phenolics, flavonoids, AVAs, and phytosterols, and whether it exerted antioxidant and anti-inflammatory effects, as well as the capacity to improve relevant intestinal parameters (pH and β-GA activities) in an AOM/DSS-induced CRC mouse model.

## 2. Materials and Methods

### 2.1. Oat Seed Germination

Oat (*Avena sativa* L.) seeds of the “Turquesa” variety were donated by Instituto Nacional de Investigaciones Forestales, Agrícolas y Pecuarias (INIFAP) Campo Experimental Bajío, Celaya, GT, MEX. For the germination process, seeds (100 g) were soaked in 1.5% sodium hypochlorite (1:6 *w*/*v*) for 30 min at 30 °C. Then, seeds were drained and washed with distilled water until they reached a neutral pH. Afterwards, seeds were soaked in distilled water (1:6 *w*/*v*) for 12 h with occasional shaking. Finally, hydrated seeds were placed in trays where a wet filter paper was extended, and then covered. The trays were introduced into a germination chamber, and filter paper was watered daily; chamber temperature and relative humidity (RH) were set at 25 °C and 60% RH, respectively. Germination was performed in darkness for five days and the experiment included three replicates. Based on previous studies, these conditions proved to be effective in ensuring the germination of the oat seeds. At the end of the process, the germination percentage was determined based on the total number of fully emerged seedlings, and the radicle length (mm) was measured using a Vernier caliper. Afterwards, oat sprouts were manually cleaned up from impurities and soil contaminants, and were immediately dried at 50 °C for 12 h, ground in a mill, and passed through a mesh with a particle size of 0.5 mm. Finally, the sprouted oat (SO) flours were stored at 4 °C until the analyses.

### 2.2. Chemical Analysis (Proximate Analysis) and Phytic Acid Content

The Official Methods of Analysis of the Association of Official Agricultural Chemists (AOAC) were used to determine the moisture (method 925.10), crude fat (method 920.85), protein (method 920.87), ash (method 923.03) [24], and crude fiber [25] contents of SO flours. The carbohydrate content was calculated by difference. The insoluble and soluble fiber contents were determined by a Megazyme β–Glucan Assay Kit Mixed Linkage (Megazyme kit, Wicklow, IRL). Insoluble and soluble fiber contents are expressed as a percentage. The phytic acid content was determined according to the method of Latta and Eskin [26] and expressed as a percentage. All determinations were performed according to three independent experiments, which included mean values of three technical repetitions.

### 2.3. Methanolic Extraction and Quantification of Total Phenolic Compounds (TPCs)

TPCs were quantified in the SO flour samples according to Singleton and collaborators [27]. Briefly, extractions with methanol/water (50:50, *v*/*v*) acidified with HCl (pH 2) and acetone/water (70:30, *v*/*v*) were combined and used for the quantification of TPC using the Folin–Ciocalteu procedure. The results of the TPCs were expressed as mg of gallic acid equivalents (GAE)/g of SO flour.

### 2.4. Abundance of Phenolic Compounds by Ultra–Performance Liquid Chromatograph (UPLC)–Mass Spectrometre (MS)

The polyphenolic extract previously mentioned was concentrated and assessed by an ultra–performance liquid chromatograph (UPLC) coupled to a photodiode array (PDA) detector and a quadrupole time-of-flight (QTOF)–MS with an atmospheric pressure electrospray ionization (ESI) (Vion, Waters Co., Milford, MA, USA) interphase for the identification of the compositions of polyphenols and AVAs. The polyphenol extract was resuspended in 1 mL of the mobile phase (95% acidified water with 0.1% formic acid and 5% of acetonitrile acidified with 0.11% formic acid) and filtered (Polyvinylidene fluoride syringe filters, 0.2 µm). Samples were kept at 10 °C in the automated injector system and injected (1 µL) into an Acquity BEH column (100 × 2.1 mm, 1.7 µm) at 35 °C under gradient conditions. The ESI–QTOF–MS conditions were as follows: Data acquisition, negative ionization mode (ESI-); mass range, 100–1200 Da; capillary voltage, 2.0 kV; cone voltage, 40 eV; collision energy, 6 V (low) and 15–45 V (high); source temperature, 120 °C; desolvation gas (N_2_), 450 °C at 800 L/h; cone gas flow, 50 L/h; lock mass correction, leucine enkephalin (50 pg/mL) at 10 µL/min.

UPLC-QTOF analysis was made as previously reported by Rodríguez–González and collaborators [28]. Peak identity was established by comparison of the exact mass of the pseudomolecular ion (confirmation through molecular composition with a mass error <10 ppm, isotopic distribution, and fragmentation pattern). Mass spectra were analyzed in comparison with the following libraries: Phenol-Explorer, Food Database, and PubChem.

### 2.5. Animals and Treatments

Male CD-1 mice (3–4 weeks of age) were purchased from the Institute of Neurobiology, Campus UNAM-Juriquilla (QT, MEX). The experiments on animals were performed in accordance with the Animal Care and Use protocol and were approved by the Ethics Committee of the Autonomous University of Queretaro (Project identification code: CBQ18/069, approved: 19 June 2018). Animals were housed in metallic cages (two or three mice per cage) and maintained under controlled conditions (12 h dark/light cycles at 25 °C and 50% ± 10% RH). Mice had free access to water and a standard pellet diet (rodent diet 501, LabDiet, USA). After two weeks of acclimatization, mice were randomly assigned to four experimental groups. Group 1: Normal (*n* = 10); group 2: AOM + DSS as control group (*n* = 10); group 3: AVA + AOM + DSS (*n* = 8); group 4: SO + AOM + DSS (*n* = 8). Animals in groups 1 and 2 were intragastrically fed with a vehicle (200 μL of saline solution). Mice in groups 3 and 4 were gavaged every morning with the phenolic-AVA extract (0.084 mg GAE/day) and 30 mg/day of SO (equivalent to 0.084 mg of phenolic extract), respectively, dissolved in 200 μL of saline solution as a vehicle, throughout the 16 week period, with an exception at week 6. After four weeks, animals in groups 2–4 were intraperitoneally injected with AOM (10 mg/kg; dissolved in NaCl 0.9%); a week later, animals received DSS (2% in the drinking water) ad libitum for seven days. Mice were weighed weekly; stool samples of 24 h were collected at week 15. Animals in all groups were euthanized by guillotine decapitation after a 16 week experimental period. Immediately after decapitation, the blood of each animal was collected, mixed by inversion to prevent clot formation, and centrifuged. The plasma and erythrocytes were separated, and erythrocytes were lysed. Afterward, the colons of the rats were immediately excised, and cecal and colonic contents were collected separately, immediately frozen, and stored at −70 °C for pH and β-GA analyses. The livers were also removed, cleansed with saline solution, and frozen with liquid nitrogen. All samples and organs were stored at −70 °C until their utilization for the corresponding analysis.

### 2.6. Assay of β-Glucuronidase Activity (β-GA) and pH in the Cecal, Colonic, and Fecal Contents

Cecal and colonic contents, as well as feces, were suspended in high–performance liquid chromatograph (HPLC) water (relation 1:5 *w*/*v*) and sonicated with icing for 3 min at 4 °C. Samples were centrifugated at 500 g for 15 min at 4 °C, and the supernatants were used for β-GA and pH values according to Jenab and Thompson [29]. β-GA was measured at 540 nm, and the amount of phenolphthalein released was determined using a phenolphthalein standard curve. β-GA is expressed as μg phenolphthalein per hour per g content.

### 2.7. Macroscopic and Histopathology Analyses

Colons were rinsed with saline solution, opened longitudinally, divided into proximal and distal sections, and inspected for macroscopic pathological lesions. Macroscopic lesions were cut and fixed in 10% buffered formalin, embedded in paraffin blocks, and processed for subsequent hematoxylin and eosin (H&E) staining and tumor classification according to Astler and Coller [30]. Lesions of the colon were classified as normal, inflammations (grade: +, ++, and +++), or adenocarcinomas [31]. The histopathological analysis of colonic lesions also consisted of the classification of infiltrating lymphocytes, presence of eosinophils, calceiform cells, muscularis externa, necrosis area, and mitosis percentage as a strategy to further characterize colonic inflammation in all groups [31,32,33,34]. The histopathological examination was performed by two observers independently. Differences of one or more grades were re-examined by both observers before reaching a final consensus.

### 2.8. Erythrocyte-Reduced Glutathione (GSH) Level

GSH content in erythrocytes was measured according to Ellman’s procedure [35]. First, erythrocytes were lysed and sulfosalicylic acid (5%) was added as a protein precipitation agent; then, samples were centrifuged, and the supernatants were assayed for GSH content. Protein concentration was determined by the bicinchoninic acid (BCA) protein assay kit (Pierce Thermo Scientific) using bovine serum albumin (BSA) as the standard. Levels of GSH are expressed as µM of GSH per mg protein.

### 2.9. Phase 2 Enzyme Assays

Total glutathione *S*-transferase (GST) and NAD(P)H:quinone oxidoreductase 1 (NQO1) activities in colonic and hepatic cytosolic fractions were measured according to the methods by Habig and collaborators [36] and Prochaska and collaborators [37], respectively. Protein concentration was determined by the BCA assay using BSA as standard. Enzymatic activities are expressed as nmol of product per min per mg protein. 

### 2.10. Statistical Analysis

Results are expressed as means ± standard error (SE), except for the chemical and nutraceutical characterization of SO and oat seed data, which are expressed as means ± standard deviation (SD). Statistical significance for chemical composition and nutraceutical compounds of SO was determined by Student’s test at *p* < 0.05. For the in vivo study, statistical significance was determined by ANOVA followed by Tukey’s test at *p* < 0.05. The Chi-square test was used to determine differences in the macroscopic and histopathological classifications of colonic lesions. Correlations were assessed by Pearson correlation analysis at *p* < 0.05. Statistical analyses were performed using JMP version 11.0.0 (Systat Software, Inc., San José, CA, USA).

## 3. Results

### 3.1. Characterization of Chemical Composition and Nutraceutical Compounds of Oat Seeds after the Germination Process

An increase in phenolic compounds, as well as changes in chemical composition, was demonstrated during the germination of grains. Therefore, the proximal compositions and nutraceutical determinations of SO flour and oat seeds are shown in Table 1; in addition, a measurement of phytic acid was carried out after five days of germination in darkness at 25 °C/60% RH. Under this condition, we reached 100% of germination and a radicle length of 6.47 ± 0.22 cm. Protein and lipid contents, as well as moisture, were higher in SO, whereas carbohydrate and ash contents were lower after oat seed germination at 25 °C/60% RH. Similarly, total fiber was also lower in SO, and soluble fiber was most affected by the germination process; more importantly, phytic acid, an antinutritional compound present in oats, was 10 times lower in oat sprouts.

As expected, TPC was higher in the germination condition (25 °C/60% RH) compared with that of the raw seed. Therefore, we aimed to identify some of the phenolic families present in the methanolic extract of SO by UPLC-MS.

### 3.2. Phytochemical Profile Induced after Germination of Oat Seeds at 25 °C/60% RH

This study identified polyphenolic families previously reported in the literature, such as AVAs, saponins, hydroxybenzoic acids, flavonols, and flavones. In addition, families not previously reported in the literature in oat grains and SO were identified here, which include mainly isoflavones, lignans, and phytosterols.

Table S1 (Supplementary Material) shows the phytochemical profiles of oat seeds and SO. Firstly, SO exhibited four major AVAs: AVA-D, AVA-L, AVA-G/1c/2p isomer I, and AVA-B, with AVA-D being the most abundant. As for the family of saponins, avenacoside A was identified in both whole oats and germinated seeds. (Epi)-catechin isomer II and (Epi)-catechin hexose isomer II were the major flavanols in SO. On the other hand, apigenin apiosyl-hexoside and luteolin apiosyl-malonyl-hexoside were identified as the two major flavones, whereas low levels of apigenin glucuronide, luteolin 7-O-hexoside, and hydroxyluteolin were detected in SO. Kaempferol pentoside-hexoside-rhamnoside was the flavonol most abundantly detected in SO, whereas quercetin dihexoside, quercetin xyloside, kaempferol, and quercetin acetyl-hexoside-rhamnoside were present with lower abundance. 

We identified members of the hydroxybenzoic acids family in raw seeds and SO that was not previously reported in the literature, such as gallic acid hexoside isomer I, hydroxybenzoic acid isomer II, and benzoic acid, while protocatechuic acid was only detected in SO. Acetylgenistin and acetyldaidzin were the two main isoflavones in SO, whereas hydroxydihydrodaidzein isomer II was only detected in SO with the lowest abundance. Acetoxypinoresinol and secoisolariciresinol were identified as the major lignans. It is important to note that there are no previous reports in which the lignin family has been identified in oat seeds. On the other hand, the most abundant phytosterols were β-campesterol hexoside and β-sitosterol hexoside; conversely, β-campesterol was only detected in SO with the lowest abundance.

### 3.3. Effect of Sprouted Oat (SO) and Its Phenolic AVA Extract (AVA) on Body Weight and Intestinal Parameters in CD–1 Mice Induced with AOM and DSS

In order to evaluate the chemopreventive efficacy of the sprouted oat (SO) and its phenolic AVA extract (AVA) in an AOM/DSS model, body weight was monitored weekly and pH values and β-GA activity were determined in cecal, colonic, and fecal samples (Table 2). At the end of the 16 week experimental period, the Normal and AOM + DSS-treated groups showed similar body weight gains. Therefore, no statistical differences were found in the final weights among the experimental groups (*p* > 0.05). These results suggest that, under experimental conditions, the carcinogenic and promoting agents (AOM + DSS), as well as the SO and AVA treatments, did not cause adverse effects related to nutrient absorption or animal growth. However, we observed anal discomfort in some mice from the 14th week of induction with AOM + DSS. In the AOM + DSS control group, four animals had rectal bleeding and five animals developed anal prolapses; meanwhile, only two animals from the AVA group and three from the SO group developed anal bleeding, and only one from each group developed anal prolapse. From week 15, bloody feces and diarrhea were more frequently observed in the AOM + DSS control group compared to those in AVA- and SO-treated groups. Animals with these conditions were individually assigned in cages and evaluated by a veterinarian on a routine basis. Regarding the survival of the animals under study, Normal and AOM + DSS control groups showed a survival rate of 100%, while AVA- and SO-treated groups showed an 89% survival rate with the death of one animal in each group, which, according to the autopsies performed by the veterinarian, were due to causes other than cancer (i.e., lack of the development of the frontal incisors and bodily injury in the metabolic cage).

As we previously reported, increased β-GA and pH values have been related to the carcinogenic effect of the AOM in the presence of DSS [38]. Therefore, β-GA and pH values in fecal, cecal, and colonic samples collected before and during the euthanizing of the animals were also determined, and the results are shown in Table 2. Although not statistically different, all AOM + DSS-treated groups had higher pH values in cecal, colonic, and fecal samples, with the values of the SO + AOM + DSS group most similar to those of the Normal group. As expected, the level of β-GA in the cecal, colonic, and fecal samples of the AOM + DSS control group was statistically higher than those of the Normal group (*p* < 0.05). Interestingly, both AVA and SO treatments significantly reduced β-GA levels in cecal and colonic samples, with the highest effect occurring in colonic β-GA values of the SO-treated groups (36% lower in comparison with that of the AOM + DSS control group).

### 3.4. Anticarcinogenic Effect of Sprouted Oat (SO) and Its Phenolic AVA Extract (AVA) on the Macroscopic and Histopathological Quantitative Classification of Colonic Lesions Induced with AOM and DSS in Male CD–1 Mice

The lesions found in the colon of each of the animals were classified macroscopically as flat-type lesions, called early lesions or plaques, and more advanced lesions, called polyps or tumors. The latter are defined as protuberances of cells that protrude into the intestinal lumen, which are characterized by increased cell division, and can be benign and asymptomatic lesions or capable of evolving into malignant lesions [39,40].

Table 3 shows the results derived from the early lesion and tumor counts found in mice of the experimental AOM + DSS model. The Normal group had an early lesion incidence of 10%, and no tumors were developed in animals in this group (*p* < 0.05). On the other hand, AVA- and SO-treated groups had the highest incidence of early lesions (100%) compared to that of the AOM + DSS control group (60%); however, the AOM + DSS control group showed the highest tumor incidence (80%) of all AOM + DSS-treated groups.

In addition, the distribution of the lesions (flat-type lesions and tumors) was also analyzed. As expected, most of the plaques and tumors in AOM + DSS-treated groups were found in the distal portion of the colon (80–100%) and, according to tumor classification, polyps in the AOM + DSS control group were mostly of the sessile type, followed by pedunculate-type polyps. Interestingly, both tumor types were reduced in SO- and AVA-treated groups; moreover, animals in the SO-treated group developed only pedunculate polyps.

**Figure 1:**
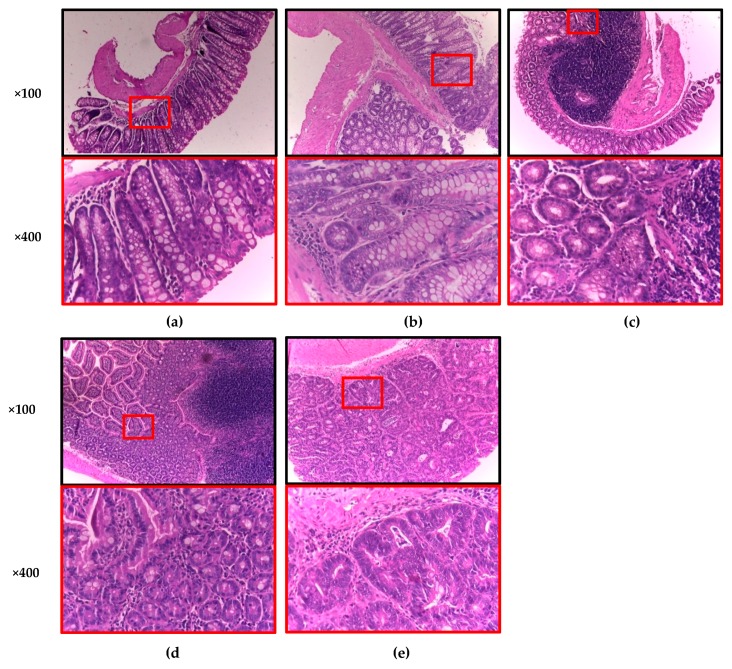
Histopathological examination of colonic mucosa of azoxymethane (AOM) + dextran sulfate sodium (DSS)-treated mice. Hematoxylin and eosin (H&E) staining: (**a**) Normal tissue; (**b**) low-grade inflammation (+); (**c**) medium-grade inflammation (++); (**d**) high-grade inflammation (+++); (**e**) adenocarcinoma. *n* = 8–10 mice per group.

The histopathological study also included analyses of the inflammation grade and dysplasia, as well as the incidence of adenocarcinomas; however, only inflammation grade and adenocarcinomas were considered (Table 4, Figure 1). The Chi-square test revealed a significant difference (α = 0.05) with respect to the incidence of adenocarcinomas. As expected, none of the animals in the Normal group developed adenocarcinomas; however, one of them (10%) presented a medium inflammation grade (++). In the AOM + DSS control group, 80% of the animals developed adenocarcinomas, and 20% showed an inflammation grade of +++. Mice in the SO + AOM + DSS group exhibited the lowest adenocarcinoma and inflammation incidence (38%); however, the phenolic AVA extract did not exert the same protection, with the incidences of adenocarcinomas and inflammation being 63% and 25%, respectively.

**Figure 2:**
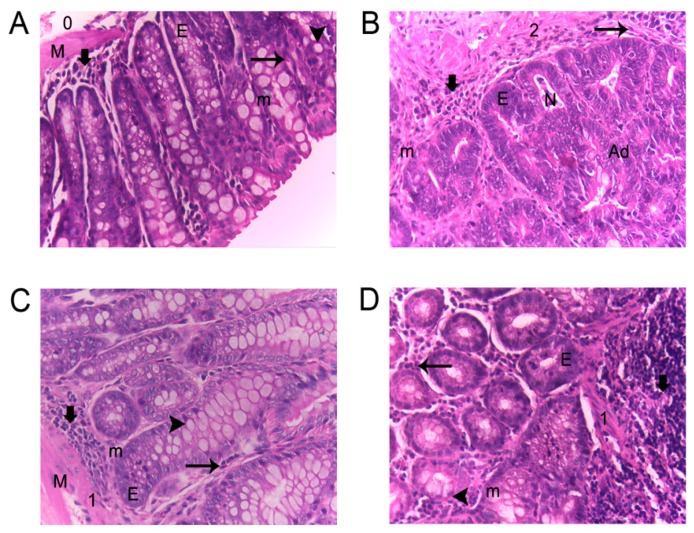
Histopathological features of colonic mucosa of AOM + DSS-treated mice. H&E staining: (**A**) Normal group, (**B**) AOM + DSS group, (**C**) AVA + AOM + DSS group, (**D**) SO + AOM + DSS group. *n* = 8–10 mice per group. (
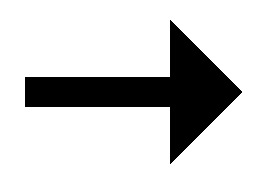
) Lymphocyte infiltration; (
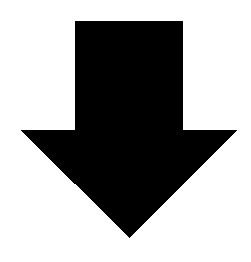
) eosinophils; (
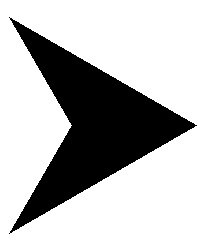
) goblet cells; (E) epithelial ridges; (M) defined muscularis; (m) mitosis; (N) necrosis; (0,1,2) inflammation grade; and (Ad) adenocarcinoma.

As inflammation was still present at the end of the experimental period (16 weeks), a quantitative and descriptive classification of the damage in the colon based on lymphocyte infiltration, eosinophils, calceiform cells, epithelial ridges, necrosis, and mitosis presence in tissues of the colon was carried out as a strategy to characterize colonic inflammation (Table 4, Figure 2). In this sense, AVA- and SO-treated groups showed a similar behavior: Medium lymphocyte infiltration, the presence of eosinophils and epithelial ridges, few calceiform cells, an absence of necrosis, and <20% of mitosis. Conversely, the AOM + DSS group showed high lymphocyte infiltration, the presence of eosinophils and necrosis, fewer calceiform cells, an absence of epithelial ridges, and 20% of mitosis.

### 3.5. Antioxidant Effects of Sprouted Oat (SO) and Its Phenolic AVA Extract (AVA) in the AOM/DSS Model

A widely accepted mechanism in cancer chemoprevention by dietary phytochemicals is through the induction of antioxidant and cytoprotective systems, such as reduced glutathione (GSH), and the activity of the phase 2 enzymes GST and NQO1, among others, through the activation of Nrf2 (NF-E2-related factor 2) signaling pathways [41]. Regarding the serum concentration of GSH, the level of GSH was significantly increased (twofold) in animals induced with AOM + DSS compared to that of animals in the Normal group (Table 5). It is important to highlight that SO and AVA treatments normalized serum GSH levels in the animals of both groups. In addition, hepatic and colonic GST activities were significantly higher in the AOM + DSS control group compared to those in the Normal group. Similarly, AVA and SO treatments achieved normalization of GST and NQO1 activities in the colon, whereas the SO + AOM + DSS group had the highest hepatic GST and NQO1 activities.

## 4. Discussion

Numerous epidemiological studies have demonstrated the relationship between high consumption of whole grains (90 g or three servings per day) and the reduced risk of coronary artery disease, cardiovascular disease, total cancer, and all-cause mortality [42]. Similarly, the suboptimal intake of whole grains (38 g/d) was associated with CRC burden across 16 European countries [43]. Therefore, an optimal intake of 50–100 g/d was considered in our study to establish the dose administered in the AOM/DSS-induced CRC mouse model (75 g/d). In addition, the germination of cereal seeds, such as oats, is a technique that has been used for centuries to soften the structure of the grain, improve its nutritional value, and reduce the antinutritional effects, while enhancing the phenolic profile with biological activity [17,44,45]. In this regard, five days of germination in darkness at 25 °C/60% RH proved to be effective in ensuring the germination of Turquesa oat seeds. Under these conditions, changes in chemical composition were expected, since total protein and lipid contents were slightly increased during a five-day germination period due to their remobilization to the developing embryo [45,46]; however, carbohydrate degradation is very limited during germination, despite the fact that starch-degrading enzymes are synthesized [47], which agrees with our results. Dietary fiber, with roughly 60% as insoluble fiber and 40% as soluble fiber [48], also decreases significantly during germination, since the release of gibberellin, a hormone capable of activating the enzyme β-glucanase, promotes β-glucan degradation, among other polysaccharides [17,49], which would explain the decrease in soluble fiber in our sample. Oats also contain other compounds such as phytic acid (5.6–8.7 mg/g; 0.56–0.87%) [9], which has its content decreased by 15%–35% during even a short three-day germination due to activation of phytase activity [50]. Although high doses of phytic acid inhibit the absorption of metals and minerals in humans, it has been observed that, in small doses, it can function as a protective factor in several chronic degenerative diseases [51]; therefore, current research only seeks to reduce the content of this antinutritional compound in various cereals.

Since part of our objective was to understand the chemopreventive effect of oats following oral administration of sprouted oat (SO) or its phenolic AVA extract (AVA), increased TPC after germination was an important keynote for determining whether the rich-phenolic extract mediated the health benefits of oats. In this regard, a more than four-fold increase in TPC was obtained after five days of germination in darkness at 25 °C/60% RH. Similar results have been previously reported [17,45,52]. This result could also reflect the better extractability of phenolic compounds from kernel structures after germination [17].

As we mentioned before, we aimed to identify the families of polyphenols contained in our sprouted oat flour from the Turquesa variety in order to inquire about the reported effects of these compounds, in addition to identifying compounds not previously reported in other oat varieties. Studies about the phytochemical profiles of oat sprouts are limited in comparison with those for whole seeds and hulks. The phenolic compounds reported in oats include phenolic acids, flavonoids, and AVA [53], with the most abundant being N-(3′,4′-dihydroxy-(E)-cinnamoyl)-5-hydroxyanthranilic acid (AVA-C or 2c), N-(4′-hydroxy-3′-methoxy-(E)-cinnamoyl)-5-hydroxyanthranilic acid (AVA-B or 2f), and N-(4′-hydroxy-(E)-cinnamoyl)-5-hydroxyanthranilic acid (AVA-A or 2p). In this study, we identified AVA-D as the most abundant AVA, followed by AVA-L, which had not been reported as one of the three most abundant AVAs in other oat varieties. In addition, of the three most abundant AVAs previously reported, only AVA-B had a higher abundance in the germination condition, confirming previous reports that indicate that AVA concentration depends on variety, fraction, genotype, and the growing environment conditions [54,55,56]. Generally, sprouts contain measurable amounts of flavonoids such as catechin and epicatechin [57]. In this regard, we identified some isomers in SO; (Epi)-catechin isomer II was the major flavanol. Other individual phenolic compounds present in SO were organic acids (hydroxybenzoic and hydroxycinnamic); the ellagic acid was detected in major abundance in SO, followed by benzoic, hydroxybenzoic isomer II, and gallic acids. For the hydroxycinnamic acids, coumaric acid isomer II was the main moiety; ferulic acid and its hexoside, as well as the feruoylquinic acid isomer I, were also present in an abundant proportion. Aborus and collaborators [16] also identified these phenolic compounds in sprouted oats of the Golozrni and Jadar varieties, with *p*–hydroxybenzoic and sinapic acids being their principal acids; however, in the Turquesa oat variety, we additionally identified hexoside compounds, isoflavones, and phytosterols. In the same report, myricetin and kaempferol were the principal flavonoids, while in our study, hexoside forms of both compounds were predominant in the Turquesa sprouted oat variety. Oksman-Caldentey and collaborators [50] reported an increase in sterols of up to 20% during the germination process; however, in this study, we observed a reduction of around 15% of these compounds. On the other hand, Pecio and collaborators [58] reported the identification of avenacoside A in different oat seeds; similarly, we identified this saponin in the Turquesa oat variety and in its SO flour.

We used the combination of AOM and DSS to induce colorectal cancer over a short-term period in mice in order to evaluate the chemopreventive effect of whole oat sprouts (SO) or their phenolic AVA extract (AVA). The AOM + DSS-treated groups predominantly developed tumors in the distal zone of the colon, which is consistent with previous reports [39,40,59,60]. In this regard, tumor (macroscopic quantitative evaluation) and adenocarcinoma incidences (histopathological classification) induced by AOM + DSS were significantly correlated (r = 0.97, *p* < 0.05), confirming that these protuberant-type lesions were capable of developing into adenocarcinomas [2,60]. The fact that SO- and AVA-treated groups developed mostly pedunculate polyps is of major relevance, since sessile polyps are considered to have greater malignancy because they have a broad implantation base, without stems, on the surface of the mucosa, so that the degeneration of the cells reaches the base earlier, whereas pedunculate polyps represent a lower malignancy because the damage to the cells takes time to reach the support base [41]. Regarding flat-type lesions, animals treated with SO and its phenolic AVA extract behaved as expected, since a plaque is an early lesion of colonic mucosa that is not considered malignant [2,30,59]. Similarly, the medium inflammation grade and the low plaque incidence in the Normal group is due to the susceptibility of the strain to spontaneously developing these types of lesions [30].

Although the animals in the AOM + DSS-treated groups did not show any signs of colitis at the end of the experimental period, we still observed inflammation grades + and ++ in the colons of animals from AVA- and SO-treated groups, which were lesser grades in comparison with that of the AOM + DSS control group (+++). According to the literature [11,12,13], in our study, we observed a mild anti-inflammatory effect of the SO and AVA treatments. It has been reported that DSS administration in the drinking water triggers a state of chronic intestinal inflammation by binding to medium-chain-length fatty acids present in the mouse colon, inducing disruption of the colonic epithelial barrier [61]. In addition, DSS causes bloody diarrhea, ulcerations, and heavy infiltration of inflammatory cells into the mucosa [62], suggesting that inflammation is involved in the tumor-promotion activity of DSS. Similar results have been observed in our study and by others in the AOM/DSS-induced CRC mouse model [38,39,40,63,64]. 

Studies regarding the chemopreventive effect of oat sprouts and whole oats are limited in comparison with those discussing the unique polyphenolic alkaloids—AVAs—exclusively extracted from oats, among other compounds such as steroidal saponins, β-glucan, and flavonoids [5,9,10,11,13,15,44,45,52,65,66,67,68], mainly through mechanisms related to their antioxidant, anti-inflammatory, immunomodulatory, antiproliferative, proapoptotic, cancer cell growth, and senescence control activities. These studies suggest that the notion of effective antitumor activity arising from whole oats may be due to the synergistic effects of multiple compounds rather than any one nutrient or compound alone. In this regard, Wang and collaborators [69], by using the 1,2-dimethyl hydrazine (DMH)/DSS mouse colon cancer model, evaluated the preventive effect of whole-oat-containing diets. Their results indicated that low-, middle-, and high-dose whole oat diets (75, 150, and 225 g/kg of experimental diets, respectively) significantly reduced the number of aberrant crypt foci (ACF) and colon tumor incidence by 60%, 100%, and 100%, respectively, in comparison with DMH/DSS-induced ICR mice (with 60% tumor incidence), and significantly suppressed colon tumor growth in vivo. According to their dosage, the low-, middle-, and high-dose whole oat diets corresponded to 60, 120, and 180 mg of whole oats per mouse per day. Furthermore, here, we report the anticarcinogenic activity of sprouted oat (SO, 30 mg/day) and its phenolic AVA extract (AVA, 0.084 mg GAE/day) in the AOM/DSS-induced mouse colorectal carcinogenesis model by reducing the adenocarcinoma incidence by 52.5% and 21.3%, respectively. Overall, the results obtained in our study suggest that, despite the fact that inflammation was still presented in colonic samples of treated groups, compounds present in SO and its phenolic AVA extract could be blocking, inhibiting, or delaying the carcinogenesis process, particularly during the stage from promotion to transformation, which is where early lesions (plaques) evolve into neoplastic lesions that can become malignant [39,40,63]. Therefore, SO and AVA treatments were effective in reducing tumor and adenocarcinoma burdens, the compositions of resident inflammatory cells, and other inflammatory scores in the colons of AOM + DSS-treated mice, suggesting that oat products still retain their biological properties even after germination processing. 

The absorption, bioavailability, and metabolism of several phytochemicals is a crucial factor in determining their biological activity against colon cancer. Fernaández-Ochoa and collaborators [23] examined at the in situ level the absorption, bioavailability, and metabolism of the phenolic compounds present in a rosemary leaf extract with proven antiproliferative and cytotoxic properties on colon cancer cells, and identified the main flavonoids, diterpenes, and triterpenes of the rosemary extract in gastrointestinal liquid and plasma samples together with metabolites from reactions of carnosic acid, carnosol, and rosmanol. In this body of evidence, it is hypothesized that the metabolites of flavonoids are primarily responsible for the observed anti-cancer effects owing to the unstable nature of the parent compounds at neutral or alkaline pH (pH > 8, as in the intestine) and their degradation by colonic microflora. Sankaranarayanan and collaborators [70] demonstrated the ability of the A-ring flavonoid metabolite, 2,4,6-trihydroxybenzoic acid (2,4,6-THBA), to inhibit Cyclin-Dependent Kinase (CDK) activity and cancer cell proliferation in colon cancer cell lines. Similarly, Wang and collaborators [20] investigated the biotransformation of AVA-C (2c) by mice and the human microbiota and found that AVA-2c and its major metabolite dihydroavenanthramide-C (M6) are bioactive compounds against human HCT-116 colon cancer cells through mechanisms related to apoptosis. On the other hand, the main mechanism involved in colon cancer prevention by oat β-glucan is the modulation of colon microbiota, which reduces the conversion of primary bile acids to secondary bile acids that are known to be tumorigenic. In addition, oat β-glucan administration increased the population of *Lactobacillus* and *Bifidobacterium*, but decreased the number of *Enterobacteriaceae* and induced a significant decrease in β-GA in rats. Moreover, oat β-glucan promotes the synthesis of short-chain fatty acids (SCFA), which are well-known anticarcinogenic compounds by colonic anaerobic bacteria and facilitate tumor cell apoptosis [9,10].

Colonic pH and β-GA activity are other physiological parameters involved in colorectal carcinogenesis. The increase in the pH of the colon and the activity of the β-GA enzyme is related to the specific colon carcinogen DMH and its metabolite AOM, as well as in the presence of the DSS promoter [39,60,63,71]. Therefore, inhibition of β-GA in cecal, colonic, and fecal samples of both SO- and AVA-treated groups is of major relevance, since β-glucuronidase is an enzyme also present in the human colonic microbiota which has the ability to hydrolyze many glucuronide conjugates and, as a consequence, can release active carcinogenic metabolites into the intestinal lumen [60]. In this regard, our results showed a positive correlation between β-GA cecal activity and the mean number of tumors and adenocarcinomas (r = 0.98 and r = 095, respectively, *p* < 0.05), which further supports the evidence that the activity of β-GA is involved in the development of colon cancer [72]; thus, modulation of β-GA through dietary treatment is confirmed as an efficient strategy for the prevention of this pathology. 

It has been reported that increases in oxidative stress and/or decreases in antioxidant capacity are involved in the development of noncommunicable diseases such as cancer [42,73,74]. As a cellular defense mechanism, the Keap1 (Kelch-like ECH-associated protein 1)-Nrf2 (NF-E2-related factor 2) system was identified to respond to redox-disrupting stimuli, which directly modify Keap1, leading to inactivation of the Keap1 function, stabilization, and nuclear translocation of Nrf2, as well as induction of cytoprotective genes, such as those for GSH synthesis, oxidative stress elimination, detoxification, drug excretion, and anti-inflammatory response, among other functions [74]. In this regard, mounting evidence suggests that the increase of the antioxidant defense system, both by enzymatic and non-enzymatic means, has been reported in cancer tissues from patients and tissue samples from in vivo models of CCR, thus indicating an augmented defense against oxidative and inflammatory damage in cancer [73,75,76,77]. In our study, similar results were observed for serum GSH levels and GST and NQO1 enzymatic activities in the colons and livers of AOM + DSS-treated mice. In fact, AOM is a procarcinogen that undergoes oxidative metabolism in the liver, generating the production of active carcinogenic electrophiles (diazonium ion) that are released into the circulation and that eventually lead to peroxidation of plasma lipids and red blood cells (erythrocytes) [78]. In this regard, GSH is the main cellular defense against oxidative stress, and its antioxidant function is based on its ability to eliminate free radicals, reduce peroxides, and participate as a co-substrate in the activity of GSH-dependent enzymes, such as GST and glutathione peroxidase, among others [79]. As reported by Matić and collaborators [80], a lower serum concentration of GSH in AVA- and SO-treated animals, as compared to those of the AOM + DSS-treated group, may be due to the increased turnover of GSH in order to prevent oxidative damage, suggesting that GSH might have been used as an antioxidant to eliminate free radical and metabolite products of AOM/DSS that are conjugated with GSH before excretion to counteract lipid peroxidation and normalize oxidative stress in the bloodstream. Furthermore, in this study, a positive correlation was found between the content of GSH in erythrocytes and the means of polyps and adenocarcinomas (r = 0.99 and r = 0.97, *p* < 0.05, respectively), which indicates that endogenous antioxidant defense mechanisms are closely related to the incidence of early lesions and the development of adenocarcinomas in colon cancer. 

Similarly, AVA and SO treatments achieved normalization of GST and NQO1 activities in the colon, suggesting that both treatments could neutralize the effects of oxidative stress at the colon level, initially generated by the oxidative metabolism of AOM and inflammation induced with DSS and, subsequently, by persistent inflammation in this disease model [75,76,81,82], resulting in a reduced carcinogenic impact. In the liver, AVA treatment normalized GST and NQO1 activities, while SO induced both activities. These results suggest that SO is more efficient in activating the Keap1-Nrf2 signaling pathway compared to treatment with AVA, which confirms that oat phenolic compounds together with β-glucans may be acting synergistically, thus offering greater protection for cancer prevention and treatment [65,83]. Tissue differences in GST activity might be attributed to cis elements in the promoter regions of these genes, known as the antioxidant/electrophile response elements, and the presence of the various transcription factors (members of the mammalian cap’n’collar (CNC) transcription factor family that possess a well-conserved basic region-leucine zipper (bZIP) motif) that heterodimerize with Nrf2, thus resulting in higher basal and inducible activities of GST in the liver as those compared to other tissues [74,84]. 

As mentioned above, the metabolism of flavonoids by gut microbiota is a crucial factor in determining their biological activity against colon cancer. Quercetin glycosides can be metabolized by intestinal bacteria into ring-fission products; the 3,4-dihydroxyphenylacetic acid (DOPAC) has recently been identified as the most active phenolic acid derived from quercetin glycosides, in terms of free-radical scavenging and induction of drug metabolizing enzymes. DOPAC simultaneously stimulates nuclear translocation of Nrf2 and aryl hydrocarbon receptor (AhR), both of which are responsible for the expression of phase I and II drug-metabolizing enzymes, such as GST and NQO1 [85]. Similarly, the oat-bran-derived phenolics ferulic and caffeic acids activate the Nrf2 signaling pathway [86,87].

Overall, the sprouting of seeds promotes degradation of macronutrients and antinutritional compounds and the biosynthesis of secondary metabolites with potential health benefits. These changes impact the nutritional value and health-promoting potential of edible seeds, such as those observed for germinated seeds and their derivatives of brown rice (*Oryza sativa*), rough rice, barley (*Hordeum vulgare*) foodstuff, and soybeans (*Glycine max* L.), from which in vivo and in vitro activities have been evaluated in order to determine their roles in CRC chemoprevention [88,89,90,91,92,93]. Here, we also provide evidence related to the chemopreventive effect of oat sprouts in AOM/DSS-induced mouse CRC, highlighting germination as a promising affordable food strategy for improving the potential health benefits of grains. 

## 5. Conclusions

This work delivers information regarding the identification of the chemical families in Turquesa oat seeds previously reported in other oat varieties, their enhanced abundance differences compared to those of sprouted oats, and their association with the chemoprotective effect observed in this study. In addition, we identified chemical families not previously reported in other oat varieties that might contribute to the anticancer effect of germinated oats. More importantly, we here provide experimental evidence for a novel biological application of *Avena sativa*—Turquesa variety—sprouts in preventing colon cancer, and we identify the contribution of the sprout’s phenolic AVA extract to achieving this effect, which is a result of its antioxidant activity, reduction of inflammatory status, and improvement of colonic physiological parameters. The major relevance of our study was the superior chemopreventive effect of the sprouted oat, probably due to the synergistic effects of multiple compounds. This further supports the potential use of oat sprouts as a functional food for colon cancer prevention.

Future investigations will now focus on the identification and quantitation of the bioactive compounds at different germination conditions to enhance the phytochemical profiles and the biological activities of oat sprouts. In addition, the examination of the absorption, bioavailability, and metabolism at several levels of the chemical families present in the sprouted oat will be of major relevance in order to clarify the absorption and metabolism of sprouted oat bioactive compounds, which in turn would contribute to a fuller understanding of the mechanisms of action of these compounds against colorectal cancer.

## Figures and Tables

**Table 1:** Chemical analysis, insoluble and soluble dietary fiber, phytic acid, and total phenolic contents in oat seed and SO (Turquesa variety) after five days of germination in darkness at 25 °C/60% relative humidity (RH).

Sample	Protein ^1^	Carbohydrates ^2^	Lipids ^1^	Ash ^1^	Moisture ^1^
Oat seed	8.83 ± 0.23 ^a^	73.37 ± 1.1 ^a^	4.41 ± 0.20 ^a^	3.99 ± 0.30 ^a^	9.4 ± 0.7 ^a^
SO	10.05 ± 0.30 ^b^	71.08 ± 0.7 ^b^	5.55 ± 0.01 ^b^	3.62 ± 0.10 ^b^	9.7 ± 0.2 ^a^
	**Insoluble Fiber ^1^**	**Soluble Fiber ^1^**	**Total Fiber ^1^**	**Phytic Acid ^1^**	**TPC ^3^**
Oat seed	55.04 ± 1.89 ^a^	18.49 ± 0.74 ^a^	73.53 ± 2.51 ^a^	0.44 ± 0.0 ^a^	0.64 ± 0.01 ^a^
SO	30.87 ± 4.23 ^b^	2.28 ± 0.52 ^b^	33.15 ± 4.65 ^b^	0.04 ± 0.0 ^b^	2.79 ± 0.06 ^b^

The results represent the average of three replicates ± standard deviation (SD). ^1^ Expressed as percentage (%) on dry basis, except for moisture. ^2^ Calculated by difference. ^3^ Expressed as mg of gallic acid equivalent (GAE)/g. TPC: Total phenolic compounds. SO: Sprouted oat. Values with different letter(s) within a column are significantly different according to Student’s test (*p* < 0.05).

**Table 2:** Changes in body weight and cecal, colonic, and fecal measurements of CD-1 mice fed with sprouted oat (SO) and its phenolic AVA extract (AVA) at week 16 of the AOM + DSS-induced colon carcinogenesis.

	Body Weight		Cecum		Colon		Feces	
Group	Initial	Final	pH	β-GA ^1^	pH	β-GA ^1^	pH	β-GA ^1^
**Normal**	30.6 ± 1.01 ^a^	39.8 ± 1.01 ^a^	7.12 ± 0.04 ^b^	3.67 ± 0.13 ^c^	7.22 ± 0.07 ^a^	3.99 ± 0.09 ^d^	7.22 ± 0.13 ^b^	4.34 ± 0.17 ^b^
**AOM + DSS control**	30.0 ± 0.76 ^a^	41.8 ± 0.79 ^a^	7.29 ± 0.04 ^a,b^	5.28 ± 0.26 ^a^	7.33 ± 0.04 ^a^	7.02 ± 0.15 ^a^	7.77 ± 0.12 ^a^	5.70 ± 0.15 ^a^
**AVA + AOM + DSS**	28.8 ± 1.03 ^a^	40.1 ± 1.10 ^a^	7.46 ± 0.05 ^a^	3.93 ± 0.08 ^b,c^	7.31 ± 0.10 ^a^	6.17 ± 0.06 ^b^	7.67 ± 0.11 ^a^	5.87 ± 0.27 ^a^
**SO + AOM + DSS**	28.9 ± 0.88 ^a^	38.9 ± 1.03 ^a^	7.13 ± 0.07 ^b^	4.11 ± 0.19 ^b^	7.09 ± 0.12 ^a^	4.50 ± 0.06 ^c^	7.62 ± 0.22 ^a^	2.51 ± 0.20 ^c^

Values are mean ± standard error, *n* = 5–10. ^1^ mg phenolphthalein per hour per g cecal, colonic, and fecal content. Values with different letter(s) within a column are significantly different according to the Tukey test (*p* < 0.05). AVA: phenolic avenanthramide (AVA) extract (0.084 mg GAE/day); SO: sprouted oat (30 mg/day).

**Table 3:** Anticarcinogenic effect of sprouted oat (SO) and its phenolic AVA extract (AVA) on the macroscopic quantitative classification of colonic lesions induced with AOM + DSS in CD-1 mice.

		Early Lesions (Flat-Type Lesions) ^1^				Tumors (Protuberant-Type Lesions) ^2^							
Group	*n*	Incidence (%)	Mean Number ^3^	Colon Distribution ^4^		Incidence (%)	Mean Number ^3^	Colon Distribution ^4^		Classification ^5^			
				Proximal	Distal			Proximal	Distal	P	S	EX	EN
**Normal**	10	10 *	0.10 ± 0.10 ^b^	1 (100%)	0 (0%)	0 *	0.00 ± 0.00 ^b^	0 (0%)	0 (0%)	0	0	0	0
**AOM + DSS**	10	60	1.00 ± 0.30 ^a,b^	0 (0%)	10 (100%)	80	4.20 ± 1.01 ^a^	0 (0%)	42 (100%)	14	16	2	10
**AVA + AOM + DSS**	8	100	2.13 ± 0.30 ^a^	0 (0%)	17 (100%)	50	0.63 ± 0.26 ^b^	1 (20%)	4 (80%)	4	1	0	0
**SO + AOM + DSS**	8	100	2.13 ± 0.61 ^a^	1 (6%)	16 (94%)	38	0.38 ± 0.18 ^b^	0 (0%)	3 (100%)	3	0	0	0

^1^ Macroscopic quantitative evaluation of flat-type lesions. ^2^ Macroscopic quantitative evaluation of protuberant lesions or tumors. ^3^ Total number of early lesions or tumors/mice per group. ^4^ Total number of early lesions or tumors and incidence between parenthesis. ^5^ Tumor classification: P, pedunculate; S, sessile; EX, exophytic, and EN, endophytic. * Incidence is statistically significant according to the Chi-square test (*p* < 0.05). AVA: phenolic AVA extract (0.084 mg GAE/day); SO: sprouted oat (30 mg/day).

**Table 4:** Quantitative classification of adenocarcinomas and histopathological inflammatory features in the AOM + DSS-induced carcinogenic study.

Group	*n*	Incidence (%)			Lymphocyte Infiltration ^1^			Eosinophils		Calceiform Cells ^2^			Epithelial Ridges		Defined Muscularis		Necrosis		Mitosis	Inflammation Grade		
		Normal Tissue	Inflammation	Adeno Carcinomas	L	M	H	Yes	No	F	M	I	Yes	No	Yes	No	Yes	No		+	++	+++
**Normal**	10	9 (90%) *	1 (10%) *	0 (0%) *	None			X				X	X		X			X	5%		1	
**AOM + DSS**	10	0 (0%)	2 (20%)	8 (80%)			X	X		X				X		X	X		20%	0	0	2
**AVA + AOM + DSS**	8	1 (12%)	2 (25%)	5 (63%)		X		X		X			X		Focal			X	15%	1	1	
**SO + AOM + DSS**	8	2 (25%)	3 (38%)	3 (38%) *		X		X		X			X		X			X	17%	1	2	

Histopathological quantitative classification according to hematoxylin and eosin (H&E) staining in colonic tissues (×400). Values are mean (*n* = 8–10 animals per group). AVA: phenolic AVA extract (0.084 mg GAE/day); SO: sprouted oat (30 mg/day). * Incidence is statistically significant according to the Chi-square test (*p* < 0.05). ^1^ Lymphocyte infiltration levels, L: Low, M: Medium, H: High. ^2^ Calceiform cell classification: F: Few, M: Moderate, I: Intense.

**Table 5:** Antioxidant effect of sprouted oat (SO) and its phenolic AVA extract (AVA) in the AOM + DSS-induced colon cancer model.

		Erythrocyte	Liver		Colon	
Group	n	GSH ^1^	GST ^2^	NQO1 ^2^	GST ^2^	NQO1 ^2^
**Normal**	10	1.40 ± 0.10 ^b^	411.5 ± 7.7 ^a^	36.2 ± 1.0 ^a,b^	60.6 ± 3.0 ^a^	54.6 ± 2.2 ^ab^
**AOM + DSS**	10	2.81 ± 0.43 ^a^	438.6 ± 12.3 ^b^	38.6 ± 0.9 ^a^	99.2 ± 6.6 ^b^	88.7 ± 2.3 ^c^
**AVA + AOM + DSS**	8	1.39 ± 0.15 ^b^	417.7 ± 10.0 ^a^	33.4 ± 0.8 ^b^	61.9 ± 2.1 ^a^	47.5 ± 3.3 ^a^
**SO + AOM + DSS**	8	1.34 ± 0.24 ^b^	506.3 ± 2.7 ^c^	58.7 ± 1.8 ^c^	55.8 ± 1.6 ^a^	58.4 ± 1.7 ^b^

Values are mean ± standard error, *n* = 5–10. ^1^ nmol per mg protein. ^2^ nmol product per min per mg protein. Values with different letter(s) within a column are significantly different according to the Tukey test (*p* < 0.05). AVA: phenolic AVA extract (0.084 mg GAE/day); SO: sprouted oat (30 mg/day).

## Supplementary Materials

Supplementary materials can be found at https://www.mdpi.com/2304-8158/9/2/169/s1. Table S1: Flavonoid and non-flavonoid profile of oat seed and SO obtained by the UPLC-QTOF analysis.

## Abbreviations

ACF	Aberrant crypt foci
AOM	Azoxymethane
AVA	Avenanthramides
β–GA	β–glucuronidase activity
CRC	Colorectal cancer
DMH	1,2–Dimethylhydrazine
DSS	Dextran sulfate sodium
GSH	Glutathione reduced
GST	Glutathione *S*–transferase
H&E	Hematoxylin and eosin
NQO1	NADPH:Quinone oxidoreductase 1
RH	Relative humidity
SO	Sprouted oat
TPC	Total phenolic compounds
