# Supplementary material for: Chemopreventive Effect of the Germinated Oat and Its Phenolic-AVA Extract in Azoxymethane/Dextran Sulfate Sodium (AOM/DSS) Model of Colon Carcinogenesis in Mice
Source: Foods. 2020 Feb 10;9(2):169. doi: 10.3390/foods9020169 (PMC7074527; doi:10.3390/foods9020169)
Supplement: Supplementary file 1 [file foods-09-00169-s001.pdf]

foods MDPI

Table S1. Flavonoid and non-flavonoid profile of oat seed and SO obtained by the UPLC-QTOF analysis

Family	Component name	Retention time (min)	Molecular formula	Expected mass	Observed mass	Mass error	Oat seed	SO (25 °C/60 % HR)
**Avenanthramide**	Avenanthramide D	6.19	C16H13NO4	283.0845	283.0867	7.79	LDL	6650.61 ± 2430.12
	Avenanthramide C	6.62	C16H13NO6	315.0743	315.0749	2.02	302.29 ± 0.00	568.10 ± 85.83
	Avenanthramide G/1c/2p isomer I	7.33	C16H13NO5	299.0794	299.0789	-1.74	2525.84 ± 0.00	2754.87 ± 830.21
	Avenanthramide G/1c/2p isomer II	7.34	C16H13NO5	299.0794	299.0789	-1.55	2913.04 ± 527.84	LDL
	Avenanthramide G/1c/2p isomer III	7.36	C16H13NO5	299.0794	299.0786	-2.44	870.96 ± 281.37	217.96 ± 116.27
	Avenanthramide B	7.66	C17H15NO6	329.0899	329.0899	-0.24	1297.30 ± 294.39	2606.07 ± 965.88
	Avenanthramide 2	8.09	C18H17NO7	359.1005	359.1003	-0.57	230.58 ± 33.11	665.69 ± 462.71
	Avenanthramide L	8.49	C18H15NO5	325.0950	325.0957	1.99	639.73 ± 142.23	4325.82 ± 945.61
	Avenanthramide E	9.85	C17H15NO5	313.0950	313.0933	-5.45	54.32 ± 0.00	325.56 ± 42.88
**Saponins**	Avenacoside A	10.48	C51H82O23	1062.5247	1062.5230	-1.62	575.17 ± 60.73	3632.47 ± 1525.98
**Flavanols**	(Epi)-gallocatechin isomer I	1.36	C15H14O7	306.0740	306.0730	-3.08	121.43 ± 0.00	284.77 ± 0.00
	(Epi)-gallocatechin gallate	1.38	C22H18O11	458.0849	458.0875	5.68	LDL	LDL
	(Epi)-gallocatechin isomer II	4.61	C15H14O7	306.0740	306.0712	-9.02	330.39 ± 8.79	540.39 ± 0.00
	(Epi)-catechin-(Epi)-catechin gallactoside	4.98	C36H34O15	706.1898	706.1960	8.87	LDL	LDL
	(Epi)-catechin isomer I	5.04	C15H14O6	290.0790	290.0763	-9.60	506.58 ± 23.86	547.84 ± 88.16
	(Epi)-Catechin isomer II	5.71	C15H14O6	290.0790	290.0776	-4.92	LDL	972.85 ± 0.00
	(Epi)-catechin hexose isomer I	5.89	C21H24O11	452.1319	452.1349	6.70	LDL	LDL
	(Epi)-catechin hexose isomer II	6.87	C21H24O11	452.1319	452.1350	6.99	618.12 ± 0.00	716.86 ± 138.73

Flavones	Luteolin rutinoside	5.38	C27H30O15	594.1585	594.1578	-1.13	785.00 ± 52.45	552.27 ± 195.27
	Apigenin apiosyl-hexoside	5.48	C26H28O14	564.1479	564.1461	-3.28	3702.91 ± 418.31	16616.23 ± 8385.16
	Hydroxyluteolin rhamnoside	5.54	C21H20O11	448.1006	448.0966	-8.96	863.53 ± 49.73	772.40 ± 272.13
	Luteolin apiosyl-malonyl-hexoside	5.66	C29H30O18	666.1432	666.1464	4.75	LDL	4092.88 ± 927.86
	Luteolin glucuronide	5.94	C21H18O12	462.0798	462.0785	-2.90	392.32 ± 0.00	774.23 ± 0.00
	Apigenin glucuronide	6.35	C21H18O11	446.0849	446.0859	2.17	534.82 ± 0.00	LDL
	Luteolin 7-O-hexoside	6.37	C21H20O11	448.1006	448.0997	-1.96	230.61 ± 33.84	LDL
	Luteolin malonyl-hexoside	6.73	C24H22O14	534.1010	534.1025	2.81	654.31 ± 99.04	624.61 ± 71.96
	Luteolin diglucuronide	6.83	C27H26O18	638.1119	638.1150	4.87	1669.21 ± 156.93	1619.69 ± 39.64
	Luteolin	7.80	C15H10O6	286.0477	286.0456	-7.53	LDL	LDL
	Hydroxyluteolin	7.86	C15H10O7	302.0427	302.0398	-9.51	220.73 ± 37.48	LDL
	Apigenin hexoside	8.30	C21H20O10	432.1056	432.1041	-3.49	3852.83 ± 0.00	2929.47 ± 0.00
	Apigenin diglucuronide	8.39	C27H26O17	622.1170	622.1161	-1.46	1111.17 ± 101.81	1064.14 ± 44.60
	Luteolin apiosyl-hexoside	8.41	C26H28O15	580.1428	580.1392	-6.21	330.27 ± 1.27	294.16 ± 128.18
	Apigenin	8.66	C15H10O5	270.0528	270.0523	-2.10	LDL	LDL
	Apigenin dihexoside	9.17	C27H30O15	594.1585	594.1578	-1.06	LDL	LDL
Flavonols	Quercetin dihexoside	3.15	C27H30O17	478.0747	478.0786	8.02	152.41 ± 0.00	LDL
	Kaempferol acetyl-hexoside	3.53	C23H22O12	490.1111	490.1144	6.72	LDL	LDL
	Myricetin rutinoside	5.13	C27H30O17	626.1483	626.1498	2.41	334.47 ± 0.00	284.58 ± 0.00
	Quercetin rhamnoside	5.19	C21H20O11	448.1006	448.0997	-1.93	299.22 ± 86.16	LDL
	Quercetin hexoside-pentoside-hexoside	5.48	C33H40O21	772.2062	772.2015	-6.10	276.29 ± 34.94	360.51 ± 0.00
	Kaempferol dihexoside	5.67	C27H30O16	610.1534	610.1517	-2.80	867.11 ± 90.92	665.26 ± 37.09

	Quercetin pentoside-hexoside	5.67	C27H30O16	610.1534	610.1545	1.88	LDL	811.64 ± 0.00
	Quercetin malonyl-hexoside	5.89	C24H22O15	550.0959	550.0973	2.69	LDL	1034.64 ± 0.00
	Kaempferol hexoside	5.94	C21H20O11	448.1006	448.1000	-1.27	LDL	413.79 ± 0.00
	Quercetin xyloside	6.06	C20H18O11	434.0849	434.0811	-8.85	153.62 ± 2.70	LDL
	Kaempferol trihexoside	6.33	C33H40O21	772.2062	772.2037	-3.24	442.85 ± 61.87	LDL
	Kaempferol pentoside-hexoside-rhamnoside	6.33	C34H40O21	740.2164	740.2148	-2.07	29395.08 ± 2058.54	13676.68 ± 2637.90
	Kaempferol acetyl-hexoside-rhamnoside	6.73	C29H32O16	636.1690	636.1639	-8.13	LDL	LDL
	Kaempferol malonyl-hexoside	6.73	C24H22O14	534.1010	534.1025	2.81	654.31 ± 99.04	624.61 ± 71.96
	Quercetin	7.86	C15H10O7	302.0427	302.0398	-9.51	220.73 ± 37.48	LDL
	Kaempferol	8.85	C15H10O6	286.0477	286.0469	-3.03	106.31 ± 0.00	LDL
	Kaempferide	8.97	C16H11O6	299.0556	299.0578	7.39	378.07 ± 0.00	LDL
	Isorhamnetin	9.05	C16H12O7	316.0583	316.0575	-2.65	290.39 ± 231.85	244.22 ± 0.00
	Kaempferol hexoside-rhamnoside	9.17	C27H30O15	594.1585	594.1578	-1.06	LDL	LDL
	Quercetin acetyl-hexoside-rhamnoside	10.08	C29H32O17	652.1639	652.1594	-6.98	185.22 ± 36.11	LDL
	Quercetin hexoside	11.01	C21H20O12	464.0955	464.0956	0.22	284.48 ± 81.40	274.62 ± 51.67
	Quercetin pentoside-rutinoside	11.78	C32H38O20	742.1956	742.2008	6.94	301.84 ± 0.00	267.72 ± 45.69
	Quercetin hexoside-xyloside	12.31	C26H28O16	478.0747	478.0754	1.31	LDL	282.82 ± 0.00
	Kaempferol hexoside-rhamnoside-hexoside	12.81	C33H40O20	756.2113	756.2123	1.33	LDL	LDL
	Myricetin rhamnoside	13.28	C21H20O12	464.0955	464.0997	9.14	1235.04 ± 0.00	LDL
	Kaempferol pentoside-hexoside	13.41	C26H28O15	580.1428	580.1392	-6.21	330.27 ± 1.27	294.16 ± 128.18

	Quercetin hexoside-rhamnoside	13.83	C27H30O16	610.1534	610.1560	4.30	LDL	LDL
**Hydroxybenzoic acids**	Gallic acid	1.03	C7H6O5	170.0215	170.0216	0.61	623.53 ± 162.41	871.40 ± 486.29
	Hydroxybenzoic acid isomer I	1.15	C7H6O3	138.0317	138.0320	1.98	55.46 ± 9.22	215.08 ± 58.27
	Hydroxybenzoic acid hexoside	1.15	C13H16O8	300.0845	300.0838	-2.38	133.91 ± 29.34	529.40 ± 79.81
	Dihdyroxybenzoic acid isomer I	1.15	C7H6O4	154.0266	154.0259	-4.43	LDL	104.37 ± 53.88
	Gallic acid hexoside isomer I	1.26	C13H16O10	332.0743	332.0714	-8.82	362.72 ± 15.08	564.17 ± 159.17
	Gallic acid gallate	1.39	C14H10O9	322.0325	322.0344	5.90	688.21 ± 0.00	LDL
	Dihydroxybenzoic acid hexoside	1.54	C13H16O9	316.0794	316.0801	1.96	867.19 ± 113.97	608.53 ± 194.85
	Gallic acid hexoside isomer IO	1.82	C13H16O10	332.0743	332.0734	-2.71	299.85 ± 0.00	600.95 ± 0.00
	Dihydroxybenzoic acid isomer II	1.87	C7H6O4	154.0266	154.0263	-2.05	69.48 ± 26.40	254.13 ± 98.52
	Hydroxybenzoic acid isomer II	3.17	C7H6O3	138.0317	138.0315	-1.33	62.48 ± 0.00	888.54 ± 329.17
	Syringic acid	3.21	C9H10O5	198.0528	198.0512	-8.02	600.06 ± 0.00	LDL
	Vanillic acid	4.05	C8H8O4	168.0423	168.0416	-4.14	75.97 ± 10.76	77.88 ± 7.75
	Benzoic acid	4.20	C7H6O2	122.0368	122.0370	1.55	341.22 ± 134.56	976.89 ± 272.52
	Gallic acid ethyl ester	4.41	C9H10O5	198.0528	198.0512	-8.44	209.89 ± 108.98	81.62 ± 0.00
	Dihydroxybenzoic acid isomer III	4.79	C7H6O4	154.0266	154.0265	-0.58	342.89 ± 45.68	260.96 ± 62.98
	Ellagic acid	5.60	C14H6O8	302.0063	302.0058	-1.65	2153.40 ± 15.87	2274.62 ± 47.25
	Ellagic acid acetyl-pentoside	6.43	C21H16O13	476.0591	476.0573	-3.77	2105.59 ± 0.00	LDL
	Hydroxybenzoic acid isomer III	6.64	C7H6O3	138.0317	138.0320	1.98	187.04 ± 13.91	177.20 ± 60.97
	Dihydroxybenzoic acid isomer IV	9.69	C7H6O4	154.0266	154.0255	-7.18	48.45 ± 0.00	LDL

	Ellagic acid pentoside	10.97	C19H14O12	434.0485	434.0490	1.08	269.30 ± 24.82	217.74 ± 16.77
**Hydroxycinnamic acids**	Caffeic acid hexoside	1.44	C15H18O9	342.0951	342.0919	-9.34	LDL	LDL
	Caffeoylquinic acid isomer I	2.39	C16H18O9	354.0951	354.0935	-4.51	LDL	LDL
	Coumaric acid isomer I	2.60	C9H8O3	164.0473	164.0476	1.64	LDL	118.80 ± 29.98
	Hydroxycaffeic acid	2.78	C9H8O5	196.0372	196.0375	1.57	LDL	155.64 ± 44.44
	Ferulic acid hexoside	3.55	C16H20O9	356.1107	356.1104	-0.80	674.53 ± 430.52	695.04 ± 83.15
	Coumaroylquinic acid isomer I	3.57	C16H18O8	338.1002	338.1000	-0.47	137.68 ± 22.87	LDL
	Coumaric acid isomer II	3.76	C9H8O3	164.0473	164.0474	0.43	223.61 ± 34.03	1488.99 ± 189.46
	Caffeoylquinic acid isomer II	3.81	C16H18O9	354.0951	354.0923	-7.96	90.82 ± 0.00	460.82 ± 0.00
	Coumaric acid hexoside	3.99	C15H18O8	326.1002	326.1002	-0.05	LDL	170.45 ± 15.35
	Sinapic acid	4.01	C11H12O5	224.0685	224.0676	-3.74	492.24 ± 50.56	222.28 ± 37.37
	Caffeoylquinic acid isomer III	4.05	C16H18O9	354.0951	354.0949	-0.58	LDL	209.80 ± 32.25
	Feruoylquinic acid isomer I	4.10	C17H20O9	368.1107	368.1110	0.84	613.78 ± 49.21	664.13 ± 204.62
	Caffeic acid	4.16	C9H8O4	180.0423	180.0416	-3.90	224.97 ± 40.32	174.22 ± 20.75
	Diferuloylquinic acid isomer I	4.58	C27H28O12	544.1581	544.1533	-8.70	LDL	LDL
	Coumaroylquinic acid isomer I	4.72	C16H18O8	338.1002	338.1035	9.85	LDL	LDL
	Feruoylquinic acid isomer II	5.09	C17H20O9	368.1107	368.1109	0.45	123.10 ± 0.28	137.74 ± 41.62
	Coumaric acid isomer III	5.13	C9H8O3	164.0473	164.0475	1.16	130.98 ± 25.00	240.34 ± 44.09
	Cinnamic acid	5.37	C9H8O2	148.0524	148.0524	-0.37	789.42 ± 0.00	230.01 ± 84.91
	Dicaffeoylquinic acid isomer I	5.45	C25H24O12	516.1268	516.1255	-2.42	LDL	LDL
	Ferulic acid	5.65	C10H10O4	194.0579	194.0579	0.15	197.53 ± 45.06	680.43 ± 113.28
	Isoferulic acid	6.01	C10H10O4	194.0579	194.0582	1.53	145.59 ± 16.52	261.48 ± 51.54

	Diferuloylquinic acid isomer II	6.28	C27H28O12	544.1581	544.1527	-9.82	LDL	350.60 ± 120.04
	Feruoylquinic acid isomer III	6.62	C17H20O9	368.1107	368.1119	3.24	LDL	LDL
	Caffeoyl tartaric acid	6.75	C13H12O9	312.0481	312.0483	0.41	LDL	LDL
	Coumaroylquinic acid isomer I	6.87	C16H18O8	338.1002	338.1006	1.32	472.77 ± 0.00	LDL
	Coumaroylquinic acid isomer I	7.71	C16H18O8	338.1002	338.1024	6.54	159.32 ± 1.69	182.38 ± 14.28
	Coumaroylquinic acid tyrosine	8.01	C18H17NO5	327.1107	327.1102	-1.50	LDL	LDL
	Caffeic acid ethyl ester	8.22	C11H12O4	208.0736	208.0726	-4.58	425.88 ± 0.00	133.43 ± 34.18
	Cinnamic acid glucose	9.58	C15H18O7	310.1053	310.1073	6.74	277.99 ± 0.00	233.65 ± 26.84
	Coumaroulquinic acid malic acid	9.70	C13H12O7	280.0583	280.0588	1.87	115.99 ± 15.45	135.91 ± 57.42
	Sinapoylquinic acid	10.33	C18H22O10	398.1213	398.1220	1.65	832.12 ± 45.17	747.96 ± 65.85
**Isoflavones**	Hydroxydihydrodaidzein isomer I	5.87	C15H12O5	272.0685	272.0685	-0.05	LDL	LDL
	Malonyldaidzin	6.77	C24H22O12	502.1111	502.1125	2.79	LDL	649.28 ± 308.32
	Acetylgenistin	6.84	C23H22O11	474.1162	474.1203	8.73	2154.74 ± 0.00	1995.66 ± 0.00
	Hydroxydihydrodaidzein isomer II	6.90	C15H12O5	272.0685	272.0683	-0.80	LDL	180.21 ± 35.27
	Daidzin	7.72	C21H20O9	416.1107	416.1075	-7.81	110.16 ± 0.00	LDL
	Malonylgenistin	8.35	C24H22O13	518.1060	518.1054	-1.24	654.87 ± 0.00	547.37 ± 0.00
	Hydroxygenistein	8.85	C15H10O6	286.0477	286.0469	-3.03	106.31 ± 0.00	LDL
	Acetyldaidzin	9.12	C23H22O10	458.1213	458.1251	8.39	1328.33 ± 0.00	1056.59 ± 69.29
**Lignans**	Lariciresinol-sesquilignan	6.33	C30H36O10	556.2308	556.2359	9.05	231.66 ± 0.00	LDL
	Dimethylmatairesinol	6.52	C22H26O6	386.1729	386.1762	8.46	LDL	548.09 ± 0.00
	Medioresinol	6.61	C21H24O7	388.1522	388.1557	9.09	LDL	LDL
	Isohydroxymatairesinol	7.53	C20H22O7	374.1366	374.1372	1.67	458.04 ± 83.77	317.39 ± 54.23

	Hydroxymatairesinol	7.54	$C_{20}H_{22}O_7$	374.1366	374.1343	-6.00	LDL	LDL
	Acetoxypinoresinol	7.75	$C_{22}H_{24}O_8$	416.1471	416.1499	6.81	LDL	2609.63 ± 0.00
	Syringaresinol	7.87	$C_{22}H_{26}O_8$	418.1628	418.1610	-4.23	LDL	725.32 ± 137.25
	Secoisolariciresinol	8.64	$C_{20}H_{26}O_6$	362.1729	362.1701	-7.78	1964.54 ± 66.57	2057.91 ± 55.54
	Secoisolariciresinol-sesquilignan	8.98	$C_{30}H_{38}O_{10}$	558.2465	558.2505	7.21	328.48 ± 0.00	245.48 ± 0.00
	Pinoresinol	9.20	$C_{20}H_{22}O_6$	358.1416	358.1395	-6.02	378.22 ± 0.00	445.74 ± 0.00
	Isolariciresinol	9.53	$C_{20}H_{24}O_6$	360.1573	360.1538	-9.68	LDL	LDL
	Lariciresinol	9.94	$C_{20}H_{24}O_6$	360.1573	360.1543	-8.28	1156.93 ± 0.00	LDL
	Matairesinol	10.37	$C_{20}H_{22}O_6$	358.1416	358.1428	3.13	469.68 ± 90.36	517.80 ± 40.05
**Phytosterols**	Beta-campesterol	15.25	$C_{28}H_{48}O$	400.3705	400.3667	-9.64	LDL	167.63 ± 5.32
	Beta-campesterol hexoside	22.53	$C_{34}H_{58}O_6$	562.4233	562.4211	-3.95	837.95 ± 63.03	957.45 ± 75.43
	Stigmasterol hexoside	22.58	$C_{35}H_{58}O_6$	574.4233	574.4228	-0.99	LDL	LDL
	Brassicasterol hexoside	23.86	$C_{34}H_{56}O_6$	560.4077	560.4061	-2.80	1479.97 ± 0.00	591.03 ± 39.50
	Beta-sitosterol hexoside	25.04	$C_{35}H_{60}O_6$	576.4390	576.4349	-7.11	832.00 ± 116.45	962.93 ± 117.69

Values (n = 3) are expressed as means ± standard deviation.

foods

MDPI

© 2020 by the authors. Licensee MDPI, Basel, Switzerland. This article is an open access article distributed under the terms and conditions of the Creative Commons Attribution (CC BY) license (http://creativecommons.org/licenses/by/4.0/).
